# The Commensal Microbiota and Viral Infection: A Comprehensive Review

**DOI:** 10.3389/fimmu.2019.01551

**Published:** 2019-07-04

**Authors:** Na Li, Wen-Tao Ma, Ming Pang, Qin-Lei Fan, Jin-Lian Hua

**Affiliations:** ^1^College of Veterinary Medicine, Northwest A & F University, Yangling, China; ^2^Animal Health and Epidemiology Center, Qingdao, China

**Keywords:** commensal microbiota, germ-free, antibiotics, virus, virus infectivity, antiviral immunity

## Abstract

The human body is inhabited by a diverse microbial community that is collectively coined as commensal microbiota. Recent research has greatly advanced our understanding of how the commensal microbiota affects host health. Among the various kinds of pathogenic infections of the host, viral infections constitute one of the most serious public health problems worldwide. During the infection process, viruses may have substantial and intimate interactions with the commensal microbiota. A plethora of evidence suggests that the commensal microbiota regulates and is in turn regulated by invading viruses through diverse mechanisms, thereby having stimulatory or suppressive roles in viral infections. Furthermore, the integrity of the commensal microbiota can be disturbed by invading viruses, causing dysbiosis in the host and further influencing virus infectivity. In the present article, we discuss current insights into the regulation of viral infection by the commensal microbiota. We also draw attention to the disruption of microbiota homeostasis by several viruses.

## Introduction

Emerging data suggest that the human body is inhabited by a wide range of microorganisms that are collectively referred to as the commensal microbiota. A majority of the microbiota reside in the intestine, while distinct populations can also be found on the surfaces of the mouth, skin, and urinary tract (1–3). A wealth of evidence suggests that this incredibly diverse microbial community is regulated by host genetic factors, and more importantly, environmental and dietary factors (4–6). We now know that the coevolution of the commensal microbiota and their hosts has resulted in a mutually beneficial condition in which the host can benefit from physiological, metabolic, and immunological regulations provided by the microbiota, while the commensal microbiota depends absolutely on the host for nutrient acquisition and propagation sites (7). For example, the gut microbiota has a crucial role in shaping immune development and functionality in the host, as reflected by extensive defects in the development of gut-associated lymphoid tissues, significantly smaller and fewer mesenteric lymph nodes and Peyer's patches, reduced secretory immunoglobulin A (IgA) production, and abnormal intestinal T cell development in germ-free (GF) mice (8–10). In addition, the gut microbiota helps the host break down dietary substances that are too large to be digested, a process that produces critical nutrients and energy for the host and generates active products (i.e., short-chain fatty acids, lactic acid, choline, and bile acids) that are essential for host health (11, 12).

The recent awareness of the essential role of the commensal microbiota in host health has remarkably improved our understanding of the interactions between microbiota and invading pathogens. In fact, a healthy commensal microbiota, as well as its products, is essential for protecting the host against a variety of pathogenic infections, through both direct elimination and indirect suppression, inside or outside of the gastrointestinal tract (13–16). Among the invading pathogens, viruses constitute one of the most common. During their infection processes, various viruses encounter the commensal microbiota of the hosts, making it possible that there are robust interactions between these viruses and the commensal microbiota. Indeed, a plethora of evidence has now shown that the commensal microbiota regulates and is inevitably regulated by invading viruses through a series of mechanisms, thereby yielding harmful or beneficial outcomes for the host (17–19). In the regulation of viral infection, commensal microbiota play varied and critical roles. They can promote viral infectivity through diverse mechanisms and can also exert substantial inhibitory effects on viral infection. On the other hand, a viral infection usually results in substantial perturbations in the commensal microbiota, causing dysbiosis in the host, which may in turn further affect viral infectivity. Although there have been several excellent reviews summarizing the modulation of viral infections by the commensal microbiota (17, 19–21), most of them focused only on infections by enteric viruses. In addition, none of these articles discussed commensal microbiota at sites other than the gut. Moreover, there have not been any literatures describing the effect of viral infections on the compositional and functional alterations of the commensal microbiota. In the present article, we systematically discuss the current progress concerning the modulation of various types of viral infections by the commensal microbiota. We also highlight the relevant mechanisms underlying these observations. In addition, we further describe the disruption of microbiota composition or homeostasis by viral infections and the relevant mechanisms.

## Promotion of Viral Infection by the Gut Microbiota (Summarized in Table 1)

### Direct Promotion of Viral Infection

#### Facilitating Genetic Recombination

The commensal microbiota can facilitate genetic recombination of viruses to enhance their infectivity. This is true for the poliovirus infection. Several studies have demonstrated that RNA viruses such as poliovirus benefit from the delivery of various viral genomes into a single target cell, thereby allowing the recombination of multiple viral genomes, and this process potentiates the viral progeny with enhanced environmental fitness (36–38). Using polioviruses encoding either DsRed or GFP and HeLa cells as target cells, Erickson et al. found that preincubation of these viruses with certain commensal microbiota significantly increased the percentage of DsRed and GFP dual-positive cells compared with those preincubated with PBS (22). Mechanistically, bacterial adhesion to HeLa cells was the main promoting force for viral coinfection. Importantly, by employing two types of viruses that are either sensitive to the drug guanidine hydrochloride while resistant to high temperature (Drug^S^Temp^R^) or resistant to guanidine hydrochloride while sensitive to high temperature (Drug^R^Temp^S^), the authors found that preincubation of viruses with bacteria increased recombination yields significantly, as reflected by the generation of polioviruses with a Drug^R^Temp^R^ phenotype, and that the recombination frequencies were positively correlated with coinfection frequencies (22). Collectively, these data suggest that interactions of the commensal microbiota and poliovirus prior to infection increases the possibility that a cell will be infected by two or more viruses, which further facilitates genome recombination of the viruses, thereby generating progenies with more diverse populations and with increased resistance to otherwise restrictive conditions.

**Table 1:** Promotion of viral infections by the commensal microbiota and the relevant mechanisms.

	**Mechanisms**	**Virus types**	**References**
Facilitating genetic recombination	Increasing the possibility that a cell will be infected by more than one virus	Poliovirus	(22)
Enhancing virion stability	Bacterial surface polysaccharides enhance the environmental stability of the virus	Poliovirus, reovirus	(23–25)
Stimulating lytic reactivation	SCFAs reactivate the lytic stage	Herpesvirus, Epstein-Barr virus	(26)
Driving the proliferation of target cells	Promoting the proliferation of CD300lf-expressing tuft cells in the colon	Norovirus	(27, 28)
Stimulating attachment to permissive cells	Increasing the binding of virus to PVR-expressing target cells	Poliovirus	(23, 24)
Contributing to viral replication	HBGA-expressing bacteria control viral replication	Norovirus	(29)
Inducing the production of immunoregulatory cytokines	LPS stimulates the production of IL-6, inducing IL-10 secretion	MMTV	(30–32)
Suppressing local antiviral immune responses	Inhibiting IFN-λ production and virus-specific immunoglobulin production.	Norovirus, rotavirus, retrovirus	(33–35)

#### Enhancing Virion Stability

In addition to facilitating genetic recombination, bacterial surface polysaccharides, i.e., peptidoglycan and lipopolysaccharide (LPS), can enhance virion stability through several mechanisms, which have been demonstrated mostly for poliovirus and reovirus. For example, gut microbiota depletion with antibiotics prior to poliovirus infection results in less susceptibility of mice and minimal viral replication in the intestine (23). Notably, when orally inoculated poliovirus was isolated from the lumen contents of untreated, antibiotic-treated, and germ-free mice, significantly higher infectivity was identified for poliovirus isolated from untreated mice. In addition, at temperatures above 40°C, markedly increased poliovirus stability was identified when they were preincubated with untreated feces or feces from germ-free mice that had been supplemented with certain bacteria. Importantly, the enhancement of viral stability did not necessarily require live bacteria, as UV-inactivated bacteria, as well as bacterial surface polysaccharides (LPS and peptidoglycan), significantly increased the viral yield over PBS when incubated with poliovirus. Furthermore, using a poliovirus mutant with reduced LPS-binding capacity, which was generated by a single amino acid substitution in the viral capsid protein VP1-T99K, the same group of authors found that while the mutant viruses showed similar replication, attachment, shedding, and pathogenesis with wild-type viruses following peroral inoculation, they displayed poorer environmental stability compared to their wild-type counterparts, as highlighted by the findings that mutant viruses were more unstable in feces and that an additional cycle of infection in mice aggravated this instability (24).

Consistent with the findings shown in poliovirus infection, another enteric virus, reovirus, also uses commensal microbiota or bacterial components to enhance thermostability (25). Similar to poliovirus, the pathogenesis of reovirus is also negatively affected by antibiotic treatment prior to infection (23). Mechanistically, the direct interaction of reovirus virions with Gram-negative and Gram-positive bacteria promotes the attachment to and infection of target cells at a variety of temperatures (23). It should be noted that commensal bacteria do not affect the overall number of viral capsid proteins, indicating that the bacterial effect on the reovirus is not exerted through regulating the overall number of viral capsid proteins (23). Collectively, these findings highlight the notion that interactions with commensal microbiota can increase the infectivity of viruses by enhancing virion stability.

#### Stimulating Lytic Reactivation

The lytic stages during viral infection involve viral gene expression, viral DNA replication and the production of new virions, making this stage indispensable for transmission and persistence of viruses (39). The direct promotion of viral infectivity by the commensal microbiota is also reflected by the stimulation of lytic reactivation by the commensal microbiota. Asai et al. found that short-chain fatty acids (SCFA) present in the culture fluids of oral bacteria induced the synthesis of early antigens in Epstein-Barr viruses (40). In addition, Gorres et al. used several short-chain fatty acids (SCFAs) and their inhibitors to explore the effect of SCFAs on lytic reactivation of Epstein-Barr virus and herpesvirus. Their results showed that all SCFAs that are histone deacetylase inhibitors can reactivate herpesvirus, whereas only several of these SCFAs reactivated the Epstein-Barr virus (26). As is widely reported, the production of SCFAs is the result of a complex interaction between the gut microbiota and diet (41, 42). These results demonstrated that there is likely a link between commensal microbiota and the lytic reactivation of viruses.

#### Driving the Proliferation of Target Cells

Tuft cells are a rare type of intestinal epithelial cells that are the reservoir for fecal shedding and persistence of murine norovirus (43). Similar to certain commensal bacteria that express receptors for human norovirus, tuft cells also express a functional receptor for norovirus, CD300lf, the expression of which dictates norovirus tropism and the efficient establishment of enteric norovirus infections (44). Elegant work from Virgin et al. revealed that both type-2 cytokines and the commensal microbiota are critical in governing the proliferation of tuft cells. In antibiotic-treated mice, a marked decrease in tuft cell-specific gene expression in the colon was observed, accompanied by a reduced number of tuft cells in the colon, a phenomenon that can be rescued by adding the type-2 cytokines interleukin (IL)-4 and IL-25 (27, 28).

#### Stimulating Attachment to Permissive Cells

The elegant work of Kuss et al. revealed that both Gram-negative and Gram-positive bacteria are potent enhancers of poliovirus infectivity (23). The authors used ^35^S-labeled poliovirus and HeLa cells and established an *in vitro* infection model. In this system, when poliovirus was incubated with *Bacillus cereus* before incubation with HeLa cells, the virus displayed dramatically increased infectivity and enhanced adherence to HeLa cells (23). Further work by the same group revealed that increased viral attachment to target cells was mainly mediated by the direct facilitation of viral binding to the poliovirus receptor (PVR) by bacterial surface polysaccharides (24). Consistent with this observation, pretreatment of HeLa cells with anti-PVR antibody significantly reduced the binding of poliovirus to HeLa cells, regardless of whether the virus was preincubated with LPS. Mechanistically, LPS treatment directly enhanced the PVR-binding ability of poliovirus, thereby stimulating attachment of the virus to target cells.

#### Contributing to Viral Replication

Certain types of viruses have evolved to interact with and use members of the host microbiota or their components to achieve optimal replication. Histo-blood group antigens (HBGAs) have been identified as receptors or coreceptors for human noroviruses. As reported, certain species of enteric bacteria express HBGAs (45). Jones et al. found that the binding of norovirus and HBGA-expressing bacteria determines the transmission and infection process of these viruses in their hosts, as infection of B cells by human norovirus can only be achieved with the presence of HBGA-positive enteric bacteria. Notably, the antibiotic depletion of normal enteric flora resulted in dramatically decreased virus titers, the mechanism of which presumably lay in the control of viral replication by the commensal microbiota (29). However, direct evidence for the control of viral replication by the commensal microbiota is lacking in this study.

### Indirect Promotion of Viral Infection

#### Inducing an Immunoregulatory Microenvironment

Emerging evidence suggests that a rich and diverse commensal microbiota plays an essential role in modulating the development of the host immune system, both inside and outside of the gut (46–49). This is true not only for the eliciting of effector immune responses by stimulating the production of various proinflammatory cytokines such as interferon (IFN)-γ during infection, but also for the establishment of an immunotolerant microenvironment by contributing to the generation of immunoregulatory cells such as Treg cells to maintain homeostasis (50–52). In fact, the commensal microbiota profoundly dictates the development, differentiation, and activation of colonic regulatory T (Treg) cells, which contribute to the maintenance of homeostasis against components of the commensal microbiota and innocuous food antigens (51). Therefore, it is possible that commensal microbiota-induced Treg cells and Treg cell-related cytokines limit the degrees of antiviral immune responses.

Several lines of evidence add to this idea. In a model of mouse mammary tumor virus (MMTV) infection, interactions between the intestinal microbiota and the invading MMTV led to an immune evasion pathway for the virus, as intestinal microbiota-derived LPS can be utilized by MMTV to generate an IL-6-dependent induction of the immunoregulatory cytokine IL-10, a key cytokine mediating the immunoregulatory functions of Treg cells (30). However, MMTV was rapidly lost in toll-like receptor 4 (TLR4) mutant mice, which exhibited robust antiviral cytotoxic immune responses (31). The same group further found that the interactions between MMTV and LPS could not be achieved without the expression of LPS-binding protein (LBP), as reflected by the fact that MMTV isolated from mice lacking LBPs cannot capture LPS and stimulate TLR4, thereby showing a remarkable transmission defect (32). Interestingly, binding to MMTV would dramatically potentiate the LPS stimulation of TLR4 expression and induction of IL-6 production compared to those with virus-free LPS, indicating that virus incorporation guarantees a greater immunostimulatory ability of LPS (32). Collectively, these data indicate that interactions between the commensal microbiota and MMTV foster the establishment of an immunotolerant microenvironment in the host and lead to persistent viral infection.

#### Suppressing Local Antiviral Immune Responses

In addition to fostering the generation of immunoregulatory Treg cells, the commensal microbiota also directly skews antiviral immunity by suppressing the activation of effector immune cells and by inhibiting the production of various inflammatory cytokines that are pivotal for virus elimination, thus creating a more favoring environment for viral infection. This is true for norovirus. In a murine norovirus infection model, the authors found that antibiotic treatment prevented persistent viral infection, a phenomenon that was reversed by replenishment of the commensal microbiota (33). Interestingly, antibiotics did not directly affect viral replication or prevent tissue infection but acted specifically to trigger the expression of receptors for antiviral cytokine IFN-λ and to stimulate the expression of *Stat1* and *Irf3*. In another murine model of norovirus infection, while IL-10^−/−^ SPF mice showed dramatically aggravated intestinal inflammation and mucosa damage, IL-10^−/−^ GF mice were free of epithelial barrier disruption, and transplantation of defined flora to these mice was sufficient to restore inflammatory lesions in the intestine (53).

In addition, mounting research has shown that the commensal microbiota also hinders the activation of antiviral humoral responses, mainly through regulating the production of virus-specific antibodies. In a murine rotavirus infection model, commensal microbiota elimination via antibiotic treatment or germ-free housing reduced the level of rotavirus antigen, delayed infection and decreased infectivity significantly (34). Notably, this phenotype was accompanied by a stronger antiviral humoral response, as more enhanced serum IgA, serum IgG and fecal IgA levels were observed. Consistent with these findings, antibiotic treatment results in greater maintenance of virus-specific antibody-secreting cells in the intestine. In contrast, when mice were treated with a low dose of dextran sodium sulfate to generate enhanced exposure to the microbiota, impaired production of rotavirus-specific antibodies following virus infection was identified (34). This finding is further supported by an independent study, which showed that although wild-type mice efficiently controlled endogenous retrovirus to a baseline level, mice with a defective antibody-secreting ability could not prevent viral activation and propagation (35). Importantly, this conclusion was true only when the intestinal microbiota was intact, as viral replication was clearly prevented in the host mice when these mice were kept in a germ-free condition, regardless of whether they had antibody-producing abilities or not, further supporting that the commensal microbiota promotes viral infectivity through suppressing the antiviral humoral immune response (35).

## Suppression of Viral Infection by the Gut Microbiota (Summarized in Table 2)

### Direct Suppression of Viral Infection

Because the commensal microbiota is present at sites that are used by certain viruses to gain entry into the host, it is likely that there are substantial interactions between the invading viruses and commensal microbiota that could have suppressive outcomes for viral infection. Supporting this notion, it was shown that lactic bacteria are able to reduce the infectivity of vesicular stomatitis virus through direct binding to the viruses, thereby blocking the cell internalization process of these viruses (54). In addition, *Enterococcus faecium* can prevent infection by influenza viruses upon direct adsorptive trapping of these viruses (55). Organisms of the commensal microbiota also produce various metabolites with antimicrobial effects to prevent virus infection. This is true for the inhibition of infections by influenza virus. First, it was found that commensal microbiota-derived LPS can bind to and destabilize the morphology of influenza virions, thereby decreasing the overall stability of the virus (56). Second, an extracellular matrix-binding protein produced by *Staphylococcus epidermidis*, a Gram-positive bacterium that lives in the human nasal cavity as a commensal, can stably bind to influenza virus and thus block further viral infection (57). In addition to influenza virus, the replication of herpes simplex virus (HSV)-2 can also be suppressed by commensal microbiota metabolites. For example, lactic acid, a major end product of the carbohydrate fermentation of all *Lactobacillus* species, can strongly inactivate HSV-2 in the vaginal mucosa by maintaining an acidic pH in the local environment (58). Consistent with this finding, in an *in vitro* study, it was shown that metabolites of vaginal *Lactobacillus* strains (i.e., lactic acid and hydrogen peroxide) exhibited potent virucidal activity, as highlighted by the dramatic suppression of virus replication by these substances (59). Commensal microbiota also exert their antiviral activity through bacterial components. For example, a vaginal strain of *Lactobacillus brevis*-extracted cell wall-associated component, which was resistant to high temperatures and protease digestion, potently inhibited the replication of HSV-2 in an *in vitro* model (60).

**Table 2:** Suppression of viral infections by the commensal microbiota and the relevant mechanisms.

	**Mechanisms**	**Virus types**	**References**
Direct suppression	Blocking cell internalization process	Vesicular stomatitis virus	(54)
	Adsorptive trapping of viruses	Influenza viruses	(55)
	Binding to and destabilizing virion morphology	Influenza viruses	(56)
	Binding to and blocking further infections	Influenza viruses	(57)
	Suppressing virus replication	HSV-2	(58–60)
Indirect suppression	Enhancing type I IFN signaling	Influenza virus	(61, 62)
	Promoting Th17 and Th22 responses	SIV	(63)
	Increasing antiviral activities of macrophages	Systemic lymphocytic choriomeningitis virus and influenza virus	(64)
	Promoting APC migration and T cell activation	Influenza virus	(65)
	Inhibiting IL-33-mediated immune suppression	HSV	(66)
	Stimulating TLR-mediated cellular and humoral antiviral immune responses	Influenza virus and vaccinia virus	(65, 67, 68)
	Enhancing CD8^+^ T cell activation of the infant by maternal microbiota	Vaccinia virus	(69)
	Preventing excessive inflammation and inflammation-associated pathology	Influenza virus, Sendai virus and SIV	(63, 70, 71)

### Indirect Suppression of Viral Infection

**Figure 1:**
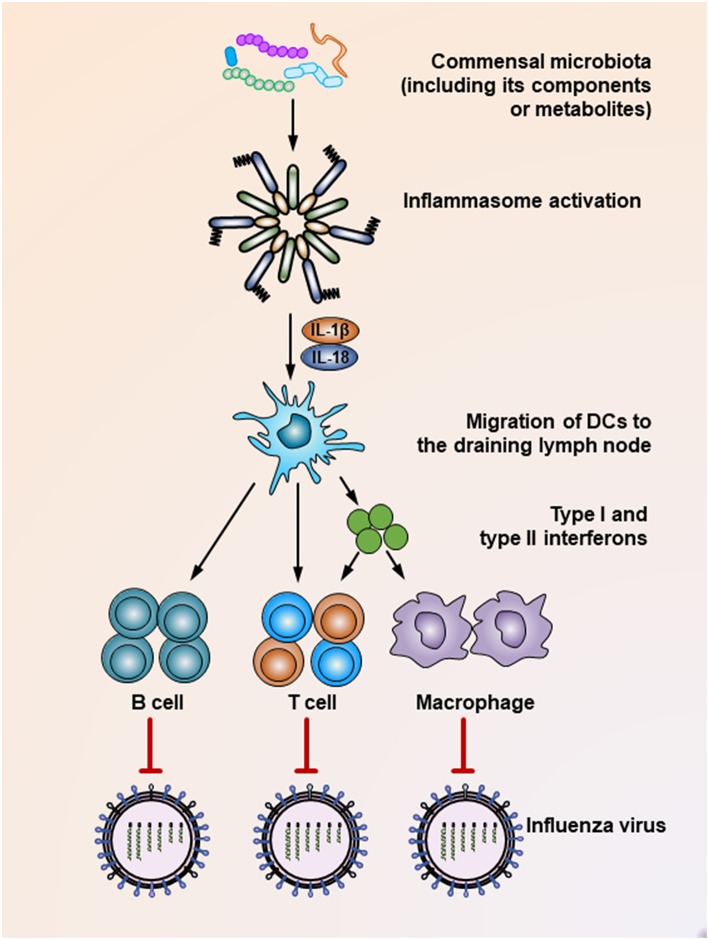
Mechanisms underlying the suppression of influenza virus infection by the commensal microbiota. During the influenza virus infection, organisms of the commensal microbiota, as well as their components (i.e., various TLR ligands) or metabolites (i.e., desaminotyrosine) activate the inflammasome, resulting in IL-1β and IL-18 production. The production of these two cytokines induces the migration of dendritic cells from the lung to the draining lymph nodes, where they act as antigen-presenting cells to prime virus-specific B cells, CD4^+^ T cells, CD8^+^ T cells, and macrophages. In addition, dendritic cells also secrete type I and type II interferons to stimulate the activation of T cells or macrophages. As a result, these effector cells secrete virus-specific antibodies or inflammatory cytokines or exert direct virus-killing effects to suppress the infection process of the influenza virus.

The commensal microbiota plays a critical role in shaping the host immune response, which essentially guarantees effective elimination of invading viruses. Supporting this notion, mounting studies have shown that intact healthy commensal microbiota help maintain robust antiviral immunity, while microbiota disruption increases viral infectivity due to the impaired capacity of the immune system to limit viral infection. For example, *Clostridium orbiscindens*, a specific human-associated gut microbe, produces desaminotyrosine to prime the amplification loop of type I IFN signaling, thereby mediating protection against influenza infection (61). In another influenza virus-infected chicken model, antibiotic treatment resulted in significantly higher oropharyngeal and cloacal virus shedding, which was also presumably mediated by reduced type I IFN responses after microbiota depletion, while the antibody-mediated antiviral immunity remained unaffected (Figure 1) (62). In simian immunodeficiency virus (SIV)-infected rhesus macaques, fecal microbiota transplantation (FMT) treatment after commensal microbiota depletion induced greater antiviral immunity, as reflected by dramatically increased peripheral Th17 and Th22 cells post-FMT (63). In contrast, when antibiotic-treated mice are infected by systemic lymphocytic choriomeningitis virus or influenza virus, their macrophages show decreased expression of genes associated with viral suppression, impaired responses to type I and type II IFNs and defective ability to control viral replication (Figure 1) (64). Consistent with this finding, it has been shown that during respiratory influenza virus infection, antibiotic exposure led to a defective generation of virus-specific CD4 and CD8 T cells and antibodies due to an impaired inflammasome-dependent migration of antigen-presenting cells (APC) from the lung to the draining lymph nodes (65) (Figure 1). This finding was further supported by another study, which showed that while oral antibiotic treatment had little effect on innate immune responses after HSV infection of the vaginal mucosa, a dramatic increase in the level of IL-33, an alarmin produced in response to epithelial cell damage, was observed after antibiotic treatment. Mechanistically, IL-33 acted as an immune-regulatory factor that suppressed local antiviral immunity by hindering the recruitment of effector T cells to the infection site and thus blocking the secretion of IFN-γ in vaginal mucosa (66).

**Figure 2:**
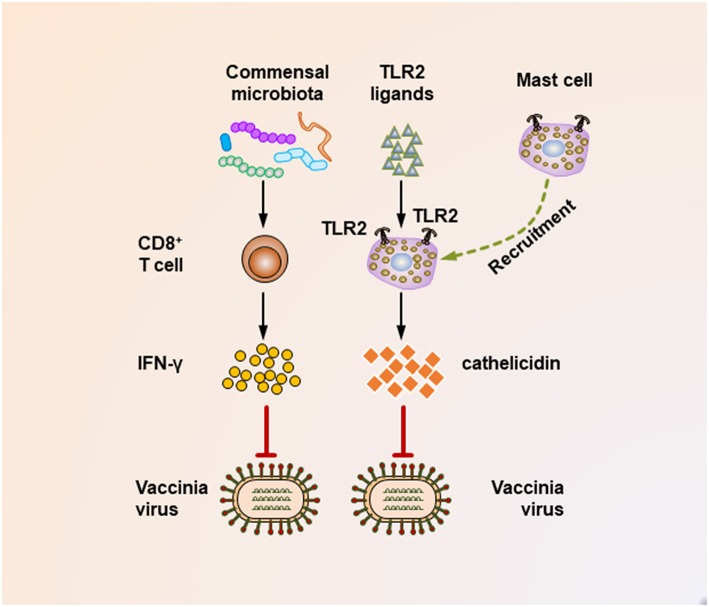
Mechanisms underlying the suppression of vaccinia virus infection by the commensal microbiota. During the vaccinia virus infection, the commensal microbiota primes virus-specific CD8+ T cells to secrete large amounts of IFN-γ, which critically mediates the corresponding antiviral immunity. In addition, during vaccinia virus infections, the activation of TLR2 by bacterial products is essential for recruiting mast cells to sites of viral infection. These mast cells also contribute to suppressing the viral infection by secreting an antiviral cathelicidin.

It seems that insufficient TLR ligand stimulation after antibiotic exposure was partly responsible for the compromised immune cell function. When TLR agonists were applied during virus challenge in antibiotic-treated mice, both cellular and humoral antiviral responses could be largely restored (Figures 1, 2) (65, 67). Moreover, TLR2 activation by bacterial products produced by the gut microbiota is necessary for the recruitment of mast cells to sites of viral infection and the further release of cathelicidin, a mast cell-derived antiviral protein (Figure 2) (68). However, this situation seems different in young mice, whose gut microbiota has not been completely established. In a hepatitis B virus infection model, TLR4-intact young mice failed to resolve viruses and developed chronic infections, while their TLR4 mutant counterparts exhibited rapid viral clearance, suggesting that an immune-tolerant pathway mediated by TLR4 signaling was predominant in young mice (72). Intriguingly, it seems that antibiotic treatment-induced gut microbiota alteration is transient and recoverable, as a more exacerbated disease condition only appears when antibiotics are used during influenza A virus infections; when such treatment ceases before the infection, neither an antiviral immunity defect, nor enhanced viral susceptibility are observed (73).

It should be noted that maternal antibiotic treatment confers an altered commensal microbiota to their offspring, thereby profoundly impacting their antiviral immunity. This idea is supported by a recent study that investigated the effect of antibiotic treatment of pregnant mice on the antiviral immunity of their neonatal offspring following vaccinia virus infection (69). In this study, maternal antibiotic treatment during pregnancy and lactation resulted in remarkable alterations in the composition of the gut microbiota of the infant mice, with *Enterococcus faecalis* predominating within the infant enteric flora. Notably, maternal antibiotic treatment resulted in an accelerated and increased mortality following vaccinia virus infection of the offspring, which was partly mediated by a defective IFN-γ-secreting ability of virus-specific CD8^+^ T cells (Figure 2).

In the case of the infection of several viruses, higher levels of immune activation may persist, associated with inflammation-induced comorbidities of the host (74). In these cases, immune recognition of the gut microbiota is necessary for the generation and activation of immunoregulatory cells to diminish local or systemic immune activation. Indeed, Rosshart et al. found that reconstitution of the gut microbiota from wild mice confers potent protective effects to laboratory GF mice during lethal influenza virus infections, an effect mainly mediated through the prevention of excessive inflammation via IL-10 and IL-13 production in the virus-affected mice by the natural gut microbiota (70). In addition, Grayson et al. found that antibiotic treatment before or during murine Sendai virus infection resulted in greatly increased morbidity and mortality, accompanied by an abnormal immune response characterized by increased proinflammatory cytokines (i.e., IFN-γ, IL-6, and monocyte chemoattractant protein 1) and decreased Tregs in the lung (71). Notably, the neutralization of IFN-γ or the adoptive transfer of Treg cells abrogated tissue inflammation and prevented increased mortality (71). In cases of human immunodeficiency virus (HIV) infection, the gut microbiota is intimately associated with activation of the immune system in HIV-infected individuals (75). Importantly, decreased activation of CD4^+^ T cells was observed post-FMT in SIV-infected rhesus macaques (63). This conclusion is reinforced by another independent study, which showed that the capacity of NKT cells to produce IL-4 and IL-10 in gastrointestinal-associated lymphoid tissues was associated with fewer markers of microbial transmission and less immune activation, a process dependent on the recognition of *Bacteroides* species by these cells (76).

Intriguingly, a recent study by Stewart et al. revealed that the interrelations between nasopharyngeal microbiota and host systemic inflammatory responses (reflected by serum metabolomic signatures) likely contribute to bronchiolitis in infants (77). Of note, the relative abundance of *Streptococcus*, which is specifically pathological in respiratory health, was positively correlated with metabolites associated with more severe disease (77). In comparison, the abundance of *Moraxella*, another important component of the nasopharyngeal microbiota, showed the opposite correlation patterns (77). Similar findings were reported in an independent study. In this study, Piters et al. showed that severity of respiratory syncytial virus (RSV) bronchiolitis induced by RSV infection was positively associated with abundance of *Streptococcus* and *Haemophilus influenzae* and negatively associated with abundance of *Staphylococcus aureus* in the nasal mucus (78). Interestingly, transcriptome profiles of whole blood from children with RSV infection and *Streptococcus*- and *Haemophilus influenza*-dominated microbiota revealed greater overexpression of several proinflammatory genes linked to macrophage and neutrophil activation (78). Thus, although the underlying mechanism is still unclear, these data clearly suggest that airway microbiota play an important role in regulating the systemic immune responses, thereby controlling the outcome of viral infections in the respiratory tract.

### Suppression of Viral Infection With Unclear Mechanisms

Several clinical cases suggest a definite suppression of viral infectivity by the commensal gut microbiota, although the detailed mechanism is unclear. For example, hepatitis B virus (HBV) e-antigen (HBeAg), which may persist in patients for years after HBV infection, is commonly used as a sensitive indicator of remission activity and improved long-term outcome (79). In a trial of FMT for the treatment of HBV, the authors found a significant decrease in the HBeAg titer in patients after FMT treatment, and the HBeAg titer decreased gradually following each FMT treatment, suggesting the efficacy of modulating the gut microbiota for chronic hepatitis B treatment (80). In another trial investigating the underlying mechanism of the development of lower respiratory tract infection (LRTI) after viral infection, the authors found that patients with a higher abundance of butyrate-producing bacteria in their fecal samples showed a 5-fold lower possibility of developing viral LRTI (81).

## Viral Infection not Affected by Commensal Microbiota

Although a wealth of evidence has shown that the commensal microbiota regulates viral infectivity, there is also evidence showing that these microbial communities may have no effects on modulating antiviral responses. For example, Gopinath et al. recently found that vaginal application of the aminoglycoside antibiotic neomycin enhanced the host resistance to a broad range of viral infections, i.e., HSVs, Zika virus and influenza A virus. However, the antiviral activity of antibiotics was independent of the commensal microbiota, as the protection was also applicable to germ-free mice and *in vitro* cultured primary cells. Instead, neomycin increased the expression of IFN-stimulated genes in the host, a process mediated by TLR3 expressed by a specific subset of dendritic cells (82). Consistent with this finding, Zhu et al. found that B cells mediate the early control of murine norovirus infections and that this effect can also be achieved even in the absence of commensal microbiota via antibiotic treatment (83). In this study, the authors found that B cells mainly function as antigen-presenting cells but not as antibody-secreting cells to exert their virus-elimination effects. In murine leukemia virus (MuLV)-infected mice, Wilks et al. found that during retrovirus infection, both the production of virus-specific antibodies and the antibody-mediated virus-neutralizing responses were independent of the commensal microbiota, as both GF and specific pathogen-free (SPF) mice produced similar levels of virus-specific antibodies, and the antibody-mediated virus-neutralizing effects were similar in both mice, suggesting that antibody-mediated immune control of MuLV does not require commensal microbiota (84). It should be noted that several earlier studies found the overall pathogenicity of murine leukemia virus were affected by the commensal microbiota. For example, compared to GF mice, conventionally reared mice developed higher levels of virus expression and longer latency period following infection of MuLV-Moloney (85, 86). However, when GF mice were stimulated with sheep erythrocytes, a significant increase in leukemia development was observed (86). The authors hypothesized that GF mice may not possess certain lymphoid cells that were required by MuLV replication stimulated by the commensal microbiota (86). Conflicting results were obtained in other studies showing that GF mice were more sensitive than conventional housed mice to MuLV infection (87). One potential explanation for this discrepancy is that the likely contamination of MuLV isolates by lactate dehydrogenase-elevating virus that can potently induce systemic lymphocyte activation (88), as suggested by Wilks et al. (89).

## Modulation of the Commensal Microbiota by Viruses

While modulation of the commensal microbiota by viruses is still poorly understood, studies to date do suggest an important role of virus infection in inducing microbiota dysbiosis. This is true for HIV/SIV infection, influenza virus infection, HBV or hepatitis C virus (HCV) infection and norovirus infection, as discussed in detail below. In addition to these four types of viral infections, alteration of the gut microbiota following infection has also been described in cases of rotavirus infection in pigs or calves (90, 91), avian leukosis viruses in chickens (92), canine distemper virus infection in giant pandas (93), and white spot syndrome virus infection in crabs (94), although the relevant reports are sporadic and the corresponding mechanistic evidence is very limited.

### HIV/SIV

A plethora of studies have emphasized that in SIV-infected non-human primates and HIV-infected patients, the commensal microbiota composition is disrupted with the enrichment of potentially pathogenic bacterial families. For example, microbial diversity in saliva of HIV patients was significantly reduced than healthy controls, accompanied by increased abundance of potentially pathogenic *Megasphaera, Campylobacter, Veillonella* and *Prevotella* species, and decreased commensal *Veillonella* and *Streptococcus* species (95, 96). In a recent study, Mukherjee et al. found that fungal communities differed significantly between HIV-infected and uninfected individuals, with *Epicoccum, Candida* and *Alternaria* being the most abundant fungi in HIV-infected individuals, while *Pichia, Candida* and *Fusarium* being the most common genera in healthy controls (97). Intriguingly, *Pichia* can efficiently inhibited *Candida* colonization (97). In bronchoalveolar lavage fluid, although there were no significant differences among the microbial composition in HIV-infected and uninfected subjects, specific metabolic profiles were associated with bacterial organisms that potentially play a role in the pathogenesis of pneumonia (i.e., Bacteria from families *Nocardioidaceae, Staphylococcaceae, Caulobacteraceae*, and genus *Streptococcus*) in HIV-infected patients (98). In a long-term monitoring of chimpanzees following SIV infection, Moeller et al. observed a marked increase in the genera *Selenomonas, Staphylococcus*, and *Sarcina*, all containing opportunistic pathogens that were never detected at high abundances in SIV-negative chimpanzees (99, 100). However, SIV infection had little effect on the frequencies of *Enterobacteriales, Bacteroidales*, or *Pseudomonas*, nor did the authors find any differences in alpha-diversity between SIV-positive and SIV-negative chimpanzees (99). In fecal samples, HIV infection was associated with consistently reduced overall microbiota richness but selective enrichment of the phyla *Firmicutes* and *Proteobacteria*, with the most prominent increase in *Bacteroides* and *arabacteroides* at the genus level (101–103). In addition, the alpha-diversity of species in the fecal microbiota is negatively associated with the severity of immunodeficiency in patients (104). Notably, combined antiretroviral therapy can effectively restore the alpha-diversity of the fecal microbiota. In addition to compositional alteration of the commensal microbiota, HIV infection also robustly alters the metabolic activity of gut microbiota (105). In contrast to healthy controls and patients with systemic lupus erythematosus and bacterium-induced diarrhea, HIV infection results in a defective metabolic capacity of gut bacteria to produce three amino acids, namely, proline, phenylalanine and lysine (105). In comparison, 3-hydroxyanthranilate, one of the major metabolites of the kynurenine pathway during the oxidative catabolism of tryptophan, was found to be significantly accumulated in the gut microbiota of all HIV-infected patients (105), which is in agreement with the finding of a previous study showing that gut microbiota with the ability to catabolize tryptophan through the kynurenine pathway are enriched in these patients (102).

Mechanistically, specific immune suppression by HIV is partly responsible for the enrichment of certain potentially pathogenic bacteria. For example, *Salmonella typhimurium*, a member of the *Proteobacteria* phylum, is tightly controlled by Th17 cells (106). In SIV-infected rhesus macaques, Th17 cells are markedly depleted, resulting in blunted Th17 responses to *Salmonella typhimurium* and finally leading to systemic dissemination of *S. typhimurium* (107), a phenomenon also observed in clinical cases of HIV-infected patients (108). In contrast, it seems that neither HIV-induced B cell dysfunction nor enteropathy affect overall systemic antibody responses to the commensal microbiota (109).

### Influenza Viruses

Influenza viruses enter the host through the upper respiratory tract (URT) and can alter the microbial composition of the URT significantly following infection. Several studies have demonstrated that influenza virus infection can result in decreased colonization by healthy bacteria and increased abundance of potentially pathogenic microbiota. For example, a case-control study using next-generation sequencing of the 16S rRNA gene to analyze specific bacteria in patients with influenza infection and healthy controls showed that the healthy core microbiota, specially *Prevotella* spp. and anaerobes, were significantly decreased in influenza virus-infected patients (110). In comparison, eight potentially pathogenic bacteria were significantly enriched in these patients, including *Haemophilus influenzae, Staphylococcus aureus, Moraxella catarrhalis, Streptococcus pneumoniae, Corynebacterium propinquum/pseudodiphtheriticum*, and *Dol osigranulum pigrum* (110). Consistent with this study, Li et al. found that *Prevotella* was decreased after H1N1 virus infection (111). It should be noted that nasopharyngeal and oropharyngeal microbiota show distinct alteration profiles following influenza infection. For example, while nasopharyngeal *Streptococcus* showed higher abundance after infection (112, 113), oropharyngeal *Streptococcus* was significantly decreased following influenza infection (112). By contrast, Ramos-Sevillano et al. found that the throat microbiota was resilient to influenza infection, with remarkably stable bacterial communities following influenza infection, which was consistent with a recent murine model-based study (114, 115). The discrepancy may result from different infection doses, sample collection method, subtypes of influenza virus and environmental factors (i.e., pH, CO_2_, and O_2_ concentrations).

In contrast to the commensal microbiota of the URT, studies have only recently begun to evaluate how influenza virus infections affect gut microbiota. Consensus has been reached that influenza virus infection alters the commensal microbiota of the host, causing corresponding disruptions of the microbiota-host homeostasis, which largely accounts for the mechanisms by which infections are established. However, this general conclusion is based on several contradictory findings. For example, while infection of influenza virus was shown to lead to an increase in *Bacteroidetes* phyla abundance in one mouse-based study (116), a remarkable drop of *Bacteroidetes* (mainly *S24-7*) was observed in influenza A virus-infected birds and mice (115, 117). Interestingly, a murine study even found unchanged *Bacteroidetes* levels after respiratory influenza virus infection (118). In addition, in avian influenza A-infected migrating whooper swans, fewer *Proteobacteria* were detected in the fecal sample (117), while this was not recapitulated in another two studies showing that selective enrichment of *Proteobacteria* (mostly *Bdellovibrionaceae*) in the gut was the result of influenza virus infection (119, 120). These contradictory findings may result from the differences in virus subtypes and doses, experimental animal types, and the age, diet, as well as the lifestyle of the same animals. However, it seems that alterations in *Firmicutes* after influenza virus infection are more uniform, as decreases in the richness of *Firmicutes* (represented by *Lactobacillus*) were observed by most of the abovementioned studies (115–117). There is also interest in whether vaccination affects commensal microbiota. However, after live attenuated influenza virus vaccination, no changes in gut microbiota composition were discovered, indicating that only live viruses can drive an altered commensal microbiota diversity (116).

Investigating the underlying mechanism, several lines of data have highlighted that the modulation of immune responses by influenza contributes to the dysbiosis of the gut. Deriu et al. found that pulmonary infection of influenza virus induced the production of type I IFNs in the lung, which acted as a central player in upregulating *Proteobacteria* and depleting obligate anaerobic bacteria (120). Moreover, IFN-mediated dysbiosis inhibited the antimicrobial inflammatory immune responses in the gut during *Salmonella* infection, further promoting *Salmonella* colonization and systemic dissemination (120). Consistent with the findings of this study, commensal microbiota dysregulation following influenza virus infection could also be the result of overproduction of a type II IFN, IFN-γ, which was secreted by a subset of lung-derived CC chemokine receptor 9 (CCR9)^+^CD4^+^ T cells in the small intestine (118). The disturbed gut microbiota further stimulated IL-15 production from intestinal epithelial cells, which subsequently facilitated the polarization of Th17 cells *in situ*, finally leading to intestinal injury (118).

### HBV/HCV

Quite a few studies have implicated that dysbiosis of the commensal microbiota occurs following infection with HBV/HCV and is relevant to the progression of liver disease. In patients with HBV, a profound alteration in the composition of gut microbiota is reflected by the significantly enriched *Actinomyces, Clostridium sensu stricto, Megamonas* and *Lachnospiraceae*, and a concomitant decrease in *Alistipes, Bacteroides, Asaccharobacter, Parabacteroides, Butyricimonas, Clostridium IV, Coriobacteriaceae, Escherichia/Shigella, Ruminococcus, Clostridiales, Enterobacteriaceae, Lachnospiraceae*, and *Ruminococcaceae* (121). In a species-level study, bacterial species with an opportunistically pathogenic nature were significantly elevated, while species with potential beneficial effects were downregulated in the fecal samples of HBV-infected patients (122). In HCV, a reduced bacterial diversity, with an increase in the order *Streptococcus* and *Lactobacillus*, and a decrease in *Clostridiales* was observed (123, 124). In another study, a lower abundance of *Firmicutes, Proteobacteria*, and *Actinobacteria* and a higher abundance of *Bacteroidetes* were detected in stool samples of HCV patients (124).

The course of liver disease progression in HBV/HCV patients can also be reflected by profiles of the commensal microbiota. In a study comparing the diversity of gut fungal microbiota in patients with HBV infection, the authors found that the diversity of intestinal fungi was positively associated with disease progression, as reflected by the higher richness of enteric fungal species in patients with HBV-related cirrhosis than in those with chronic infection. Moreover, patients with chronic hepatitis B exhibited higher richness of fungal species compared with asymptomatic HBV carriers and healthy controls (125). In a cross-sectional investigation, it was shown that alpha-diversity of the fecal microbiota decreased significantly from healthy controls to HCV patients without cirrhosis to those with cirrhosis (126). In addition, the ratio of *Bifidobacterium*/*Enterobacteriaceae* was suggested to be a sensitive biomarker for the clinical course of HBV, as a gradual decrease in this ratio was observed in asymptomatic HBV carriers, patients with chronic hepatitis B, and patients with HBV-associated cirrhosis (127).

In addition to the gut microbiota, an alteration of the oral microbiota in HBV-affected individuals has also been reported in a recent study, which showed that the ratio of *Firmicutes*/*Bacteroidetes* was increased significantly (128). Interestingly, HBV infection resulted in a marked increase in bacteria capable of producing H_2_S and CH_3_SH, implicating the potential contribution of the altered microbiota to the oral malodor in these patients (128). Compositional and metabolic changes in the tongue-coating microbiota have also been documented in HBV-infected individuals. As reported, yellow tongue was associated with higher HBV titers compared with those in patients with a white tongue. Moreover, a significant decrease in *Bacteroidetes* and an increase in *Proteobacteria* was found in HBV-associated yellow tongues, which also showed a selective enrichment of the metagenomic pathways involved in amino acid metabolism, consistent with the metabolic disorder of these patients (129).

In summary, HBV/HCV infection indeed caused profound changes in the composition and metabolism of the commensal microbiota. However, it should be noted that most of these studies are based on observational data. Thus, further studies exploring the underlying mechanism of HBV/HCV-induced microbiota alterations are clearly needed.

### Norovirus

Norovirus represents one of the most important causes of acute viral gastroenteritis worldwide (20), usually causing severe diarrhea and occasionally causing chronic infections in immunocompromised individuals (20). In a human-based study investigating the effect of norovirus infection on the gut microbiota, the authors found that while the fecal microbiota in most infected individuals exhibited a similar composition to that of uninfected controls, a significant loss of diversity and richness of the gut microbiota characterized by a clear increase in the relative numbers of *Proteobacteria* and a corresponding decrease in *Bacteroidetes* was observed in a small proportion of norovirus-infected patients (130). Further analysis revealed that a single operational taxonomic unit of *Escherichia coli* was partially responsible for the increase in *Proteobacteria* in these patients (130). Consistent with this observation, human norovirus can bind to certain human stool-isolated bacteria, including those in the phylum *Proteobacteria* (i.e., *Hafnia alvei, Citrobacter* spp., *Klebsiella* spp., and *Enterobacter cloacae*), with high efficiency, implicating a direct modulation of the gut microbiota by norovirus (131). Intriguingly, murine norovirus even has the capacity to maintain gut homeostasis and shape intestinal immunity, similar to the functions of the gut microbiota. Kernbauer et al. found that norovirus infection of antibiotic-treated or germ-free mice restored the aberrant lymphocyte compartment and the abnormal intestinal morphology without inducing overt inflammation and disease (132). Importantly, norovirus infection protected antibiotic-treated mice from dextran sulfate sodium-induced intestinal injury and *C. rodentium* superinfection, suggesting that norovirus has the potential to replace the beneficial functions of commensal microbiota in the intestine (132).

### Theiler's Murine Encephalomyelitis Virus (TMEV)

Emerging evidence supports the intriguing concept of the brain-gut microbiome axis and has shown bidirectional interactions within it (133, 134). Several systems including the central, autonomic and enteric nervous systems, the neuroendocrine and the neuroimmune systems are at work to guarantee proper functioning of this axis (135). Current data have suggested that this complex communication axis is essentially linked to the regulation of multiple aspects of host physiology ranging from gastrointestinal homeostasis to psychiatric, motivational and cognitive functions (136–139). As a result, perturbation of the brain-gut microbiome axis is involved in several disorders including metabolic dysregulation and psychiatric and non-psychiatric diseases (140, 141). Thus, it is not surprising that viral infection-associated brain abnormalities can result in gut dysbiosis, which may in turn affect the development and severity of virus-associated tissue pathology. This was evidenced by a recent study investigating the effect of intracerebral TMEV infection on commensal microbiota (142). In this study, Carrillo-Salinas et al. found that TMEV infection was associated with significantly altered gut microbiota, reflected by a reduction in the relative abundance of *Alloprevotela* (phylum *Bacteroidetes*) at 14 days post infection and a decrease in *Anaerotruncus* (phylum *Firmicutes*) and *Akkermansia* (phylum *Verrucomicrobia*) while an increase in *Clostridium XIVa* (phylum *Firmicutes*) at 28 days post infection (142). Intriguingly, the effect of TMEV infection on gut microbiota is profound and lasting, as alterations in *Firmicutes* and *Bacteroidetes* of the gut microbiota still exist at 85 days post infection (142). In addition, oral administration of antibiotics dampened TMEV infection by enhancing antiviral immune responses during acute phase of infection (142).

## Conclusions and Future Perspectives

We have discussed the current understanding of the modulation of virus infectivity by the commensal microbiota of the host and the underlying mechanisms in this regulation. We have also described the contribution of viral infection to the disturbances of microbiota homeostasis in the host. We do not yet fully understand the extent to which commensal microbiota may determine the efficiency of viral replication, transmission, and persistence, and in most cases reported, the relevant mechanisms underlying the influence of the host microbiota by invading viruses are unclear. However, the data presented do support an intimate interaction between the commensal microbiota and invading viruses, an interaction that always dictates the outcome of an infection. Thus, it is tempting to speculate that antiviral drugs aimed at modulating virus-microbiota interactions may be particularly effective in controlling the activity of many viral diseases. In fact, the pharmaceutical application of FMT and probiotic supplements have already been proven useful in reducing the severity of several diseases in human- and non-human primate-based studies, although these efforts may turn out to be ineffective in certain circumstances and may even result in unwanted complications (63, 143–146). Therefore, there are still major gaps in our understanding of the interactions between the commensal microbiota and viruses, and constant optimization of these potential treatment methods is clearly needed to better control viral infections via the modulation of commensal microbiota.

Recent works have shed light on the role of the commensal microbiota in health and many diseases. However, considering the immense diversity of the commensal microbiota, most studies investigating the functional attributes of these microbial communities are based on population-level analyses, and the majority of species in the commensal microbiota have never been isolated and cultured in the laboratory, thus greatly hindering progress in identifying the unique phenotypes and functions of each species of the commensal microbiota to minimize the risk of complications of FMT brought about by unwanted microbiota. Therefore, further efforts into developing more effective approaches for commensal microbiota culture *in vitro* are urgently needed. In addition, an important gap in microbiota research is that most studies discuss only bacterial microbiota and often overlook fungi or viruses, which are also important components of the commensal microbiota of the host (19, 147). Thus, future studies on components of the commensal microbiota other than bacteria are also needed.

As discussed above, several studies have suggested that the commensal microbiota may potently promote viral infections, and commensal microbiota depletion with antibiotics could conceivably be used as a strategy to treat viral infections. However, one must recognize that all the antibiotic-induced inhibitions of viral infection have been designed only in mouse studies, and usually only the effect of such treatment on viral infection is evaluated, regardless of the potentially negative consequences on a broader scale of infections. For example, when the majority of commensal microorganisms are depleted by broad-spectrum antibiotic treatments, the beneficial effects of the commensal microbiota on host health, the maintenance of host physiological homeostasis and the promotion of host immune functions, will concomitantly disappear, making these adverse consequences far outweigh the benefits of blocking a particular viral infection. In addition, it is now well-recognized that antibiotic overuse leads to the emergence of antibiotic-resistant bacteria or even superbacteria that may bring about severe or even life-threatening infections. Thus, we do not advocate the use of antibiotics to treat or prevent viral diseases in humans. However, understanding how the commensal microbiota enhances viral infection, especially the molecular requirements for the microbiota-mediated promotion of viral infections, may lead to the development of novel, feasible antiviral strategies.

Emerging data suggest that the newly discovered cyclic-GMP-AMP (cGAMP) synthase (cGAS)-cGAMP-stimulator of interferon genes (STING) pathway as the major pathway in sensing cytosolic DNA following viral infections (148–150). In fact, the cGAS-cGAMP-STING axis has been shown to be involved in restricting both DNA and RNA virus infections (150, 151). Notably, a recent work has shown that guanylate cyclase C, which is expressed on intestinal epithelial cells and is crucial for the generation of cGMP, critically regulates microbiome composition of the intestine through maintaining barrier integrity by cGMP production (152). However, during viral infections, the effect of cGAMP on the commensal microbiota is unclear. Considering that the mechanism underlying the modulation of commensal microbiota by viral infections has not been fully clarified, this intriguing question undoubtedly warrants further investigations.

## Author Contributions

W-TM and J-LH designed the structure of this review. NL and W-TM wrote the manuscript. NL, MP, Q-LF, and J-LH revised the manuscript. All authors have reviewed the final version of the manuscript.

### Conflict of Interest Statement

The authors declare that the research was conducted in the absence of any commercial or financial relationships that could be construed as a potential conflict of interest.
